# Implementing a micromechanical model into a finite element code to simulate the mechanical and microstructural response of arteries

**DOI:** 10.1007/s10237-020-01355-y

**Published:** 2020-06-30

**Authors:** Daniele Bianchi, Claire Morin, Pierre Badel

**Affiliations:** Mines Saint-Etienne, Univ. Lyon, Univ. Jean Monnet, INSERM, U 1059 Sainbiose, Centre CIS, 42023 Saint-Etienne, France

**Keywords:** Multiscale homogenization, Nonlinear finite element formulation, Collagen fiber rotation, Tension–inflation test

## Abstract

A computational strategy based on the finite element method for simulating the mechanical response of arterial tissues is herein proposed. The adopted constitutive formulation accounts for rotations of the adventitial collagen fibers and introduces parameters which are directly measurable or well established. Moreover, the refined constitutive model is readily utilized in finite element analyses, enabling the simulation of mechanical tests to reveal the influence of microstructural and histological features on macroscopic material behavior. Employing constitutive parameters supported by histological examinations, the results herein validate the model’s ability to predict the micro- and macroscopic mechanical behavior, closely matching previously observed experimental findings. Finally, the capabilities of the adopted constitutive description are shown investigating the influence of some collagen disorders on the macroscopic mechanical response of the arterial tissues.

## Introduction

The etiology of cardiovascular diseases is debated, and the therapeutic approaches, as well as the diagnosis, still have a high percentage of failure (Kelly and Fuster 2010). Advanced screening and effective treatments for vascular pathologies can be developed with an improved understanding of cardiovascular biomechanics and mechanobiology. Recently, computational biomechanical models have provided a novel point of view into the mechanics of biological tissues, in both diagnostic and treatment scenarios (Bianchi et al. 2017; Falcinelli et al. 2019; Morganti et al. 2019; Perrin et al. 2015). In the context of arterial tissues, the constitutive description represents a key aspect, especially for insight on the mechanisms that govern the onset of the vascular pathologies. Although several models exist to describe the mechanical behavior of the arterial tissue, they generally are based on phenomenological parameters (Holzapfel et al. 2000; Auricchio et al. 2012). This prevents from the possibility to understand if some histological features can promote a pathological behavior of the arterial tissues. Alternatively, the structurally motivated constitutive model proposed by (Marino and Vairo 2013) has been effective in describing the anisotropic and nonlinear features of arterial mechanical responses by introducing parameters that directly translate to histological and structural properties. However, this approach neglects the non-affine kinematics of the collagen fiber network within the arterial structure. In fact, several works and experimental studies (Cavinato et al. 2020; Krasny et al. 2017; Chandran and Barocas 2006; Screen et al. 2004) demonstrate the occurrence of non-affine deformations in the soft fibrous tissue. For this reason, in this work, the constitutive description of the arterial tissue integrates both the non-affine fiber kinematics, addressed via the Eshelby’s inclusion problems theory (Morin et al. 2018) and a structural approach able to recover the nonlinear features of collagen fibers (mainly de-crimping) by means of histological intrinsic parameters (Marino and Wriggers 2017). In addition, since the use of hypoelasticity has been demonstrated as a suitable tool for soft tissue biomechanics (Freed 2008, 2010; Morin et al. 2018), the constitutive description is based on a hypoelastic formulation. The work herein also develops an integrated computational procedure for nonlinear finite element (FE) analyses to extend the utility of our model into the FE framework and to analyze the nonlinear mechanical response of the arterial tissue. Moreover, the computational framework is applied to reproduce experimental tests (Krasny et al. 2018) in order to validate the model prediction in both micro- and macro-mechanical behavior. Furthermore, thanks to the adopted constitutive model, case studies addressing collagen disorder (e.g., reduction in content, morphological related changes) are investigated. Finally, the novel capabilities of the developed numerical framework are discussed.

## Modeling assumption and governing equations

**Fig. 1:**
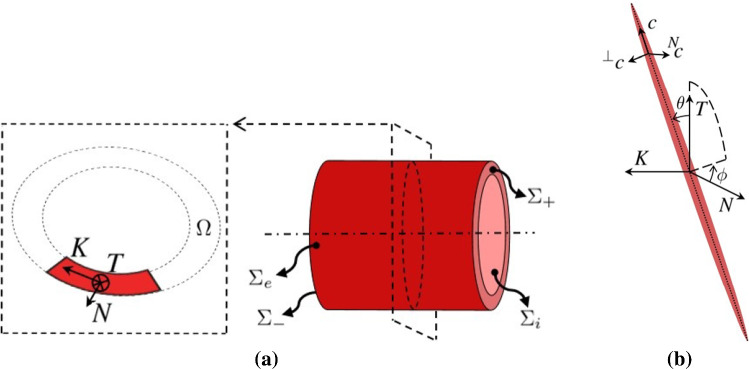
Representation of: **a** geometry of the problem with the associated local reference system (${\underline{K}}$, $\underline{T}$, $\underline{N}$), **b** the Eulerian angles defining the orientation in space of the fiber and the material reference system ($\underline{c}, {}^\perp \underline{c}, {}^{N} \underline{c}$) of the fiber-like inclusions

In this paper, the mechanical problem of tensile–inflation tests on an arterial segment is numerically addressed. The main assumptions related to this problem read as:

- the initial configuration is a hollow cylinder with length $\mathcal {L}_o$, internal radius $\mathtt {R}_o$ and thickness $\delta _o$, composed of two concentric layers, namely the adventitia as the outer layer and the media as the inner one (with respective thickness $\delta ^A_o$ and $\delta ^M_o$). The intima is not modeled due to its negligible role in the arterial mechanics. The initial configuration is considered as unloaded;
- the arterial segment is subjected to a tension–inflation loading, characterized by the application of a pressure field $P^{{\rm im}}(t)$ on the inner surface $\Sigma _i$ and of an axial displacement field $\underline{U}^{{\rm im}}(t)$ on the right transverse section $\Sigma _+$. No force is applied on the external surface $\Sigma _e$; a zero displacement is imposed on the left transverse section $\Sigma _-$ (Fig. 1a). Two loading paths are considered, namely a tensile test under a uniform and constant inner pressure and an inflation test at a constant imposed stretch;
- under the previous assumptions, the governing equations read as follows:
  1 $$\begin{aligned}&\underline{\text {div}}[ \mathbf{T} (\underline{X},t)]= \underline{0} \quad \forall \, \underline{X}\in \, {\varOmega }, \end{aligned}$$
  2 $$\begin{aligned}&\mathbf{D} {(\underline{X},t)} = \tfrac{1}{2}[\nabla \underline{V}{(\underline{X},t)}+{}^t\nabla \underline{V}{(\underline{X},t)}] \quad \forall \, \underline{X}\in \, {\varOmega }, \end{aligned}$$
  3 $$\begin{aligned}&\underline{V}{(\underline{X},t)}=\dot{\underline{U}}{(\underline{X},t)} \quad \forall \, \underline{X}\in \, {\varOmega }, \end{aligned}$$
  4 $$\begin{aligned}&\dot{\mathbf{T }}(\underline{X},t) = \mathbb {C}(\underline{X},t):\mathbf{D} (\underline{X},t)&\quad \forall \, \underline{X}\in \, {\varOmega }. \end{aligned}$$
  The boundary conditions are:
  5 $$\begin{aligned}&\mathbf{T} {(\underline{X},t)} \cdot \underline{n}\,{(\underline{X},t)} = P^{\,im}(\underline{X},t) \,\, \underline{n}\,{(\underline{X},t)} \quad \forall \, \underline{X}\in \, \Sigma _i, \end{aligned}$$
  6 $$\begin{aligned}&\mathbf{T} {(\underline{X},t)} \cdot \underline{n}\,{(\underline{X},t)} = \underline{0} \quad \forall \, \underline{X}\in \, \Sigma _e, \end{aligned}$$
  7 $$\begin{aligned}&\underline{U}{(\underline{X},t)} = \underline{U}^{{\rm im}}(\underline{X},t) \quad \forall \, \underline{X}\in \, \Sigma _+, \end{aligned}$$
  8 $$\begin{aligned}&\underline{U}{(\underline{X},t)} = \underline{0} \quad \forall \, \underline{X}\in \, \Sigma _-, \end{aligned}$$
  and the initial conditions read as:
  9 $$\begin{aligned}&\mathbf{T} (\underline{X},0)= \mathbf{D} (\underline{X},0)= \mathbf{0}&\quad \forall \, \underline{X}\in \, {\varOmega }, \end{aligned}$$
  10 $$\begin{aligned}&\underline{U}{(X,0)} =\underline{V}{(\underline{X},0)}= \underline{0} \quad \forall \, \underline{X}\in \, {\varOmega }, \end{aligned}$$
  whereby $\underline{\text {div}}$ is the divergence operator, $\nabla$ is the Eulerian gradient operator, ${}^t$ is the transpose operator, $\underline{X}$ is the macroscopic location vector, *t* is the time, $\dot{( \cdot )}$ is the material derivative of $( \cdot )$, $\underline{U}$, $\underline{V}$, $\underline{n}$, $\mathbf{T}$ and $\mathbf{D}$ are, respectively, the displacement, velocity, unit outward normal vector, Cauchy stress and Eulerian strain rate fields; $\mathbb {C}(\underline{X},t)$ is the effective hypoelastic macroscopic stiffness at point $\underline{X}$ and instant *t*;
- the stiffness tensor $\mathbb {C}$ results from the current microstructural arrangement of the arterial tissue constituents. In particular, addressing medial and adventitial layers, the mechanical response of the arterial tissue is described via a multiscale procedure which is detailed in Sect. 3. In both media and adventitia, a hypoelastic formulation is adopted neglecting dissipative mechanism;
- the presence of crimped collagen fibers in the unloaded adventitia induces a nonlinear mechanical response as a consequence of fiber de-crimping and fiber rotation. These processes are explicitly modeled in the constitutive relation of the adventitia. To this aim, different collagen fiber families are incorporated in the model, and the constitutive model takes into account their strain-induced de-crimping and rotation;
- the media are considered as purely elastic.

## Constitutive model

The constitutive response of arterial tissue is modeled via a multiscale approach coupling the non-affine fiber kinematics, addressed via nonlinear random homogenization (Morin et al. 2018), with the nonlinear behavior of collagen fibers resulting from their progressive de-crimping (Maceri et al. 2010, 2013; Marino and Wriggers 2017). The multiscale formulation links the apparent tissue stiffness to the current deformation state and to structural properties in terms of histological features. Moreover, to describe the anisotropic behavior characterizing the arterial tissue, a local reference system (${\underline{K}}$, $\underline{T}$, $\underline{N}$) is defined at each macroscopic point $\underline{X}$. In detail, the local orthonormal reference system (${\underline{K}}$, $\underline{T}$, $\underline{N}$), depicted in Fig. 1, is defined as follows:

- $\underline{N}$ is the outward unit normal to $\Sigma _e$: it defines the radial direction;
- $\underline{T}$ is orthogonal to $\underline{N}$, lying in the tangential plane to $\Sigma _e$, and parallel to the centerline direction of the arterial segment: it represents the axial direction;
- ${\underline{K}}$ is such that ${\underline{K}} = \underline{T} \times \underline{N}$ (with $\times$ as the cross product), lying in the tangent plane to $\Sigma _e$: it represents the circumferential direction.

### Medial layer

The medial layer is mainly made of elastic lamellae and smooth muscle cells. For the sake of simplicity, and since the focus of the paper is laid on the fiber kinematics in the adventitial layer, the medial layer is modeled as a homogeneous layer, having a hypoelastic stiffness $\mathbb {C}^{M}$chosen isotropic and characterized by a Young’s modulus $E^{M}$ and a Poisson’s ratio $\nu ^{M}$ (Table 1 for numerical values).

### Adventitial layer

#### At a scale of few hundred micrometers

According to multiphoton imaging and histology, the adventitial layer is composed of different fiber networks (mainly elastin and collagen) and fibroblasts being embedded in a surrounding matrix. Consequently, each point $\underline{X}$ of the previously described structure is modeled as a representative volume element (RVE) with a characteristic size $l$, being much smaller than the characteristic size of the structure $\mathcal {L}$, and much larger than the characteristic size of the heterogeneities $r^{\,c}$.

**Fig. 2:**
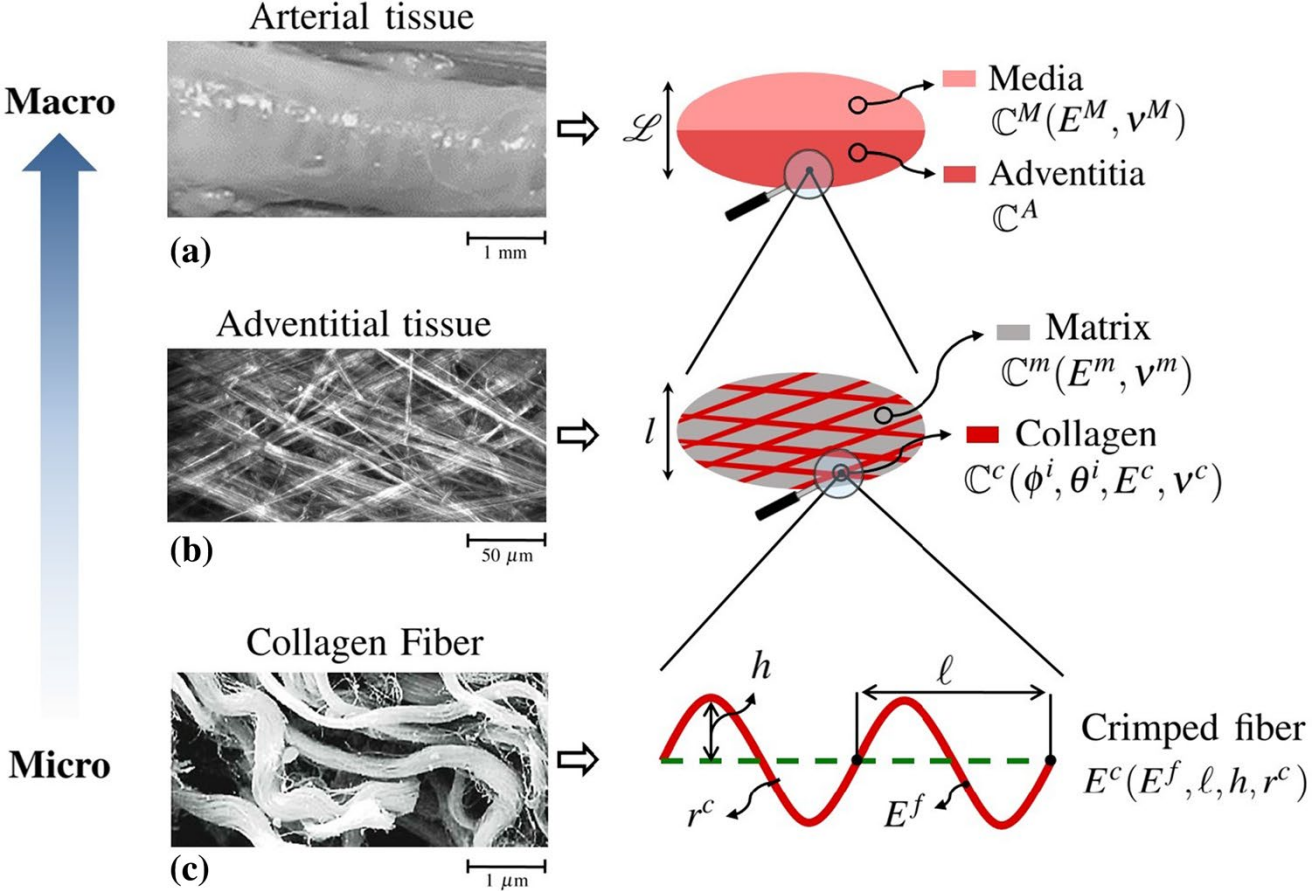
Schematic representation of micro-to-macro-homogenization rationale including a sketch of the representative volume element (RVE) of the arterial tissue. Picture **a**, **b** reproduces from Krasny et al. (2017), and picture **c** reproduces from Ushiki (2002)

For the description of the microstructure, a statistical approach is chosen and material phases are defined, namely a collagen phase, represented by infinitely long cylindrical inclusions, having a volume fraction $f^{\,c}$, and a matrix phase filling the rest of the volume and having therefore a volume fraction $f^{\,m}=1-f^{\,c}$ (Fig. 2).

With respect to the (macroscopic) local reference system (${\underline{K}}$, $\underline{T}$, $\underline{N}$), the orientation of each *i*-th fiber phase is defined through the Euler angles $\phi ^{i}$ and $\theta ^{i}$, as represented in Fig. 1. In addition, a (microscopic) reference system ($\underline{c}, {}^\perp \underline{c}, {}^{N}\underline{c}$) is defined with respect to the (macroscopic) local reference system by

11 $$\begin{aligned} {\left\{ \begin{array}{ll} \underline{c}^{\,i}=(\cos \phi ^{i} \,\cos \theta ^{i})\underline{N} + (\sin \phi ^{i}\,\cos \theta ^{i})\underline{T} - (\sin \theta ^{i})\underline{K}\\ {}^\perp \underline{c}^{\,i}=-(\sin \phi ^{i})\underline{N} + (\cos \phi ^{i})\underline{T} &{} \\ {}^{N} \underline{c}^{\,i}=(\cos \phi ^{i}\,\sin \theta ^{i})\underline{N} + (\sin \phi ^{i}\,\sin \theta ^{i})\underline{T} + (\cos \theta ^{i})\underline{K} \end{array}\right. } \end{aligned}$$

The elastin fibers are not explicitly modeled here, but are accounted for in the definition of the matrix stiffness (Table 1).

The mechanical response of the RVE is modeled in the framework of large strain continuum micromechanics, as proposed by Morin et al. (2018). In this context, the average strain rate and spin of each phase are linearly related to the strain rate $\mathbf{D}$ imposed at the boundary of the RVE via fourth-order tensors:

12 $$\begin{aligned} \mathbf{d}^{c,\,i}=\mathbb {A}^{c,\,i} : \mathbf{D}, \, \quad \mathbf{\omega }^{c,\,i}=\mathbb {R}^{c,\,i} : \mathbf{D} \end{aligned}$$

where $\mathbf{d}^{c,\,i}$ is the average strain rate tensor of the *i*-th collagen fiber family, $\mathbf{\omega }^{c,\,i}$ is the average spin tensor of the same fiber family and the fourth-order tensors $\mathbb {A}^{c,\,i}$ and $\mathbb {R}^{c,\,i}$ represent the strain rate concentration tensor and strain rate-to-spin concentration tensor for the *i*-th collagen fiber phase, respectively. The semi-analytical expressions of these fourth-order tensors can be suitably estimated from the matrix inclusion problem (Eshelby 1957), extended to the Mori–Tanaka scheme (Mori and Tanaka 1973), since the adventitial microstructure is modeled as a matrix-inclusion problem, where the interactions between the inclusions are taken into account via the strain rate seen by the matrix (for more details, the reader is referred to (Morin et al. 2018)).

The sum of the microscopic strain rate and of the spin allows to update the orientation of the fibers, which, besides the de-crimping process, are also rotating under the application of a mechanical load:

13 $$\begin{aligned} \underline{\dot{a}}= (\mathbf{{d}}^{c,\,i}+\mathbf{{\omega }}^{c,\,i})\cdot \underline{a} \quad \forall \,\, \underline{a} \, \in \, \{ \underline{c}^{\,i}, {}^\perp \underline{c}^{\,i}, {}^{N}\underline{c}^{\,i} \} \end{aligned}$$

At the microscopic scale, both the matrix and the collagen fibers exhibit a hypoelastic constitutive behavior, whereby the local strain rates and the objective stress rates are proportional:

14 $$\begin{aligned} \overset{\triangle }{\mathbf{t}}(\underline{x})=\mathbb {C}(\underline{x}):\mathbf{d} (\underline{x}) \end{aligned}$$

with $\underline{x}$ as the microscopic location vector describing position within the RVE and $\mathbb {C}$ as the location-dependent elasticity tensor expressed as a function of the phase Young’s modulus *E* and Poisson’s ratio $\nu$,

15 $$\begin{aligned} \mathbb {C}(\underline{x})= {\left\{ \begin{array}{ll} \mathbb {C}^m = \mathbb {C}^m (E^m,\nu ^m) &{} \quad \forall \, \underline{x}\in \, \text {matrix}\\ \mathbb {C}^c = \mathbb {C}^c (E^c,\nu ^c) &{} \quad \forall \, \underline{x}\in \, \text {fibers} \end{array}\right. } \end{aligned}$$

The objective Jaumann rates of the Cauchy stress are chosen, i.e.,

16 $$\begin{aligned} \overset{\triangle }{\mathbf{t}} =\dot{\mathbf{t}} + \mathbf{t} \cdot \mathbf{\omega } - \mathbf{\omega } \cdot \mathbf{t} \end{aligned}$$

Finally, the macroscopic fields are retrieved by use of stress and strain rate average rules (Morin et al. 2018). In addition, combining the macro-to-micro-relation (12), with the constitutive relation (14) and (16), and by means of the stress average rule, the homogenized stiffness of the adventitia $\mathbb {C}^{\,A}$ can be computed.

#### Collagen fiber modeling

In the adventitia, the collagen fibers, which are modeled as straight cylinders in the upper scale RVE, are actually crimped in the unloaded configuration (Fig. 2). The crimping is progressively reduced under the application of a mechanical load. A different modeling strategy is adopted in order to account for this de-crimping mechanism: neglecting any effect induced by the vessel curvature, the collagen fibers in the arterial tissue are modeled as planar homogeneous beams with a constant circular cross section with radius $r^{\,c}$ (Marino and Wriggers 2017). In agreement with histological evidence, the collagen fiber has a periodic shape modeled by a sinusoid:

17 $$\begin{aligned} g(s)= h \sin \left( \,\frac{s}{\ell } \,\right) \end{aligned}$$

with *s* as the curvilinear coordinate along the centerline, *h* and $\ell$ the sinusoid amplitude and period, respectively (Maceri et al. 2010).

The application of a mechanical load leads to the progressive de-crimping, i.e., to an evolution of the parameters $\ell$ and *h* (with $\ell _o$ and $h_o$ as initial values), as well as to a change in the axial apparent stiffness of the crimped beam. These evolutions are retrieved from the combination of a constrained Hu-Washizu variational principle and the principle of virtual work (for technical details, the reader is referred to (Marino and Wriggers 2017)). As a result, the chord elastic modulus of the collagen fiber is expressed by:

18 $$\begin{aligned} E^c=E^f I^c\langle \cos \alpha \, \rangle [I^c\langle \cos ^2\alpha \rangle +A^c\langle g^2\rangle ]^{-1} \end{aligned}$$

whereby $\langle \cdot \rangle$ denotes the curvilinear average operator defined along the curvilinear coordinate *s* following the fiber centerline, $E^f$ is the elastic modulus of the collagen fiber, $A^c=\pi (r^{\,c})^2$ is the cross-sectional area, $I^c=\pi (r^{\,c})^4/4$ is the inertial moment of the crimped fiber and $\alpha =\frac{h}{\ell } \cos \left( \,\frac{s}{\ell }\, \right)$ is the fiber centerline slope (Table 1 for their numerical values). Finally, the chord Young’s modulus $E^c$ is incorporated into the hypoelastic stiffness tensor of the collagen fibers $\mathbb {C}^c$ of the upper scale RVE together with a constant Poisson’s ratio $\nu ^c$.

## Numerical strategy

### Overall strategy

Temporal and spatial integrations are needed to solve Eqs. (1)–(10). Concerning the spatial discretization, a finite element approach is used; concerning the temporal discretization, all mechanical variables are evaluated at a series of discrete time instants $t_n$, $n \in \{ 0,...,N \}$, which are all separated by a time interval $\varDelta t$. A subscript *n* after any variable *a* denotes the time instant at which the variable is evaluated: $a(t_n)=a_n$. The applied loading is discretized accordingly. An explicit forward Euler scheme is chosen, i.e., for any quantity *a*, its time derivative reads as:

19 $$\begin{aligned} \dot{a}_{n} \, \varDelta t =\varDelta a_{n} = a_{n+1} - a_n \end{aligned}$$

Assuming that the problem is solved up to time $t_n$, the solution is then computed at the next time step $t_{n+1}$. Multiplying Eq. (1) by a virtual velocity field $\tilde{\underline{V}}$ and integrating over the structure’s volume, one gets a weak formulation, reading, at time $t_{n+1}$, as

20 $$\begin{aligned} \int _{\varOmega _{n+1}} \mathbf{T} _{n+1}: \tilde{\mathbf{D }} \,\, {\rm d}\varOmega = \int _{\Sigma _{n+1}} [\,\mathbf{T} _{n+1} \cdot \underline{n}_{\,n+1}] \cdot \tilde{\underline{V}} \,\, {\rm d}\Sigma \quad \forall \,\, \tilde{\underline{V}} \end{aligned}$$

whereby $\tilde{\mathbf{D }}$ is the virtual strain rate computed from $\tilde{\underline{V}}$. For the sake of clarity, the spatial dependence of all variables is implicit. Considering the forward Euler scheme (19), together with the constitutive Eq. (4), one gets:

21 $$\begin{aligned} \mathbf{T} _{n+1} = \mathbf{T} _n + \dot{\mathbf{T }}_{n} \varDelta t = \mathbf{T} _n + {\mathbb {C}_{n}} : \mathbf{D }_{n} \varDelta t \end{aligned}$$

with

22 $$\begin{aligned} \mathbb {C}_{n}(\underline{X})= {\left\{ \begin{array}{ll} \mathbb {C}^A_{n} &{} \quad \forall \, \underline{X}\in \, \text {adventitial layer}\\ \mathbb {C}^M_{n} &{} \quad \forall \, \underline{X}\in \, \text {medial layer} \end{array}\right. } \end{aligned}$$

Besides, the right-hand side integral of (20) is decomposed over different surfaces; when accounting for Eqs. (5) - (8), one gets

23 $$\begin{aligned} \begin{aligned} \int _{\Sigma _{n+1}} [\,\mathbf{T} _{n+1} \cdot \underline{n}_{\,n+1}] \cdot \tilde{\underline{V}} \,\, {\rm d}\Sigma&= \int _{\Sigma _{i,\,n+1}} P^{\,im}_{n+1} \,\, \underline{n}_{\,n+1} \cdot \tilde{\underline{V}} \,\, {\rm d}\Sigma \, + \\&\quad + \int _{\Sigma _{+,\,n+1} \cup \,\,\Sigma _{-,\,n+1}} [\,\mathbf{T} _{n+1} \cdot \underline{n}_{\,n+1}] \cdot \tilde{\underline{V}} \,\, {\rm d}\Sigma \quad \forall \,\, \tilde{\underline{V}} \end{aligned} \end{aligned}$$

and

24 $$\begin{aligned} \begin{aligned}&\int _{\Sigma _{+,\,n+1} \cup \,\,\Sigma _{-,\,n+1}} \underline{U}_{\,n+1} \cdot [\,\tilde{\mathbf{T }} \cdot \underline{n}_{\,n+1}] \,\, {\rm d}\Sigma = \\&= \int _{\Sigma _{+,\,n+1}} \underline{U}^{{\rm im}} \cdot [\,\tilde{\mathbf{T }} \cdot \underline{n}_{\,n+1}] \,\, {\rm d}\Sigma \quad \forall \,\, \tilde{\mathbf{T }} \end{aligned} \end{aligned}$$

which, when inserted in Eq (20), provides

25 $$\begin{aligned} \begin{aligned}&\int _{\varOmega _{n+1}} \mathbf{T} _{n}: \tilde{\mathbf{D }} \,\, {\rm d}\varOmega + \int _{\varOmega _{n+1}} \mathbf{D }_{n}: \mathbb {C}_{n} : \tilde{\mathbf{D }} \,\, {\rm d}\varOmega =\\&= \int _{\Sigma _{i,\,n+1}} P^{\,im}_{n} \,\, \underline{n}_{\,n+1} \cdot \tilde{\underline{V}} \,\, {\rm d}\Sigma \,\,+ \int _{\Sigma _{i,\,n+1}} \varDelta P^{\,im} \,\, \underline{n}_{\,n+1} \cdot \tilde{\underline{V}} \,\, {\rm d}\Sigma \,+\\&\quad + \int _{\Sigma _{+,\,n+1} \cup \,\,\Sigma _{-,\,n+1}} [\,\mathbf{T} _{n+1} \cdot \underline{n}_{\,n+1}] \cdot \tilde{\underline{V}} \,\, {\rm d}\Sigma \quad \forall \,\, \tilde{\underline{V}} \end{aligned} \end{aligned}$$

and

26 $$\begin{aligned} \begin{aligned} \int _{\Sigma _{+,\,n+1} \cup \,\,\Sigma _{-,\,n+1}} \underline{U}_{\,n+1} \cdot [\,\tilde{\mathbf{T }} \cdot \underline{n}_{\,n+1}] \,\, {\rm d}\Sigma =\\ = \int _{\Sigma _{+,\,n+1}} \underline{U}^{{\rm im}}_{n+1} \cdot [\,\tilde{\mathbf{T }} \cdot \underline{n}_{\,n+1}] \,\, {\rm d}\Sigma \quad \forall \,\, \tilde{\mathbf{T }} \end{aligned} \end{aligned}$$

whereby $\tilde{\underline{V}}$ is any virtual velocity, $\tilde{\mathbf{T }}$ any virtual surface traction in the sets of continuous and continuously differentiable, respectively, first-order tensors and second-order tensors.

This weak formulation displays two major difficulties: the unknown character of the geometrical configuration $\varOmega _{n+1}$ and the evolving character of $\mathbb {C}_{n}$ due to local rotations and de-crimping of the fibers. As a consequence of the first difficulty, the first term on the left side of equation (25) does not simplify with the first term implying the loading at $t_n$ on the right-hand side. The simplification of the terms shows an error strictly related to the discretization step size. Numerous preliminary simulations, reproducing uniaxial and biaxial stretching tests, have been performed in order to minimize the error, while maintaining a light computational cost. The results of the performed analyses show that the choice of a small enough discretization step size assures a negligible error. Accordingly, the solution algorithm neglects this difficulty for the sake of simplicity. To overcome the other difficulties, the problem is solved iteratively, by splitting the set of equation in two subsets:

- the weak form of the equilibrium equations (25) - (26) is solved by considering that the stiffness tensor remains constant over the time interval;
- the stiffness tensor is locally updated, by considering that the computed strain rate field $\mathbf{D} _{n}$ remains constant over the time interval.

### Enforcing the constitutive relations and updating the reference systems

The solution of the equilibrium equation provides a displacement increment $\varDelta \underline{U}_n$, which allows calculation of the deformation gradient as

27 $$\begin{aligned} \mathbf{F}_{\,n+1} = \varDelta \mathbf{F}_n\cdot \mathbf{F}_{\,n} \end{aligned}$$

with $\varDelta \mathbf{F}_n = \mathbf{I}+ \nabla (\varDelta \underline{U}_n)$ as the incremental deformation gradient and $\mathbf{I}$ as the second-order identity tensor. This, in turn, is used to update the macroscopic reference system as:

28 $$\begin{aligned} {\left\{ \begin{array}{ll} \underline{K}_{\,n+1} = \frac{\varDelta \mathbf{F}_n \cdot \underline{K}_{\,n}}{\Vert \varDelta \mathbf{F}_n\cdot \underline{K}_{\,n}\Vert } \\ \underline{T}_{\,n+1}= \frac{\underline{\bar{T}}_{\,n+1} - (\underline{\bar{T}}_{\,n+1} \cdot \underline{K}_{\,n+1})\, \underline{K}_{\,n+1}}{\Vert \underline{\bar{T}}_{\,n+1} - (\underline{\bar{T}}_{\,n+1} \cdot \underline{K}_{\,n+1})\, \underline{K}_{\,n+1}\Vert }\ \,\,\,\text {with} \quad \underline{\bar{T}}_{\,n+1}=\frac{\varDelta \mathbf{F}_n\cdot \underline{\bar{T}}_{\,n}}{\Vert \varDelta \mathbf{F}_n\cdot \underline{\bar{T}}_{\,n}\Vert } \\ \underline{N}_{\,n+1}=\underline{K}_{\,n+1} \times \underline{T}_{\,n+1} \end{array}\right. } \end{aligned}$$

On the other hand, each macroscopic point $\underline{X}$ undergoes an Eulerian strain rate $\mathbf{D}_n=\varDelta \mathbf{F}_n/\varDelta t$, which is applied in terms of a velocity field $\underline{v}_{\,n}(\underline{x})$ on the RVE attached to that macroscopic point $\underline{X}$:

29 $$\begin{aligned} \underline{v}_{\,n}(\underline{x})= \mathbf{D}_n \cdot \underline{x} \quad \forall \, \underline{x} \, \in \, \partial \varOmega . \end{aligned}$$

Then, the homogenization procedure runs as follows: based on the known configuration at time $t_n$, the concentration tensors $\mathbb {A}^{c,\,i}_n$ and $\mathbb {R}^{c,\,i}_n$ can be evaluated; the macroscopic average fields follow from (12) and the update of the local reference system from applying the explicit Eulerian scheme (19) to (13); finally, the updated local reference system reads as:

30 $$\begin{aligned} {\left\{ \begin{array}{ll} {\underline{c}^{\,i}}_{n+1} =\frac{{\underline{c}^{\,i}}_{n} + \varDelta ({\underline{c}^{\,i}}_{n})}{\Vert {\underline{c}^{\,i}}_{n} + \varDelta ({\underline{c}^{\,i}}_{n}) \Vert } \\ {{}^\perp \underline{c}^{\,i}}_{n+1} =\frac{{{}^\perp \underline{c}^{\,i}}_{n} + \varDelta ({{}^\perp \underline{c}^{\,i}}_{n})}{\Vert {{}^\perp \underline{c}^{\,i}}_{n} + \varDelta ({{}^\perp \underline{c}^{\,i}}_{n}) \Vert } \\ {{}^{N} \underline{c}^{\,i}}_{n+1} =\frac{{{}^{N} \underline{c}^{\,i}}_{n} + \varDelta ({{}^{N} \underline{c}^{\,i}}_{n})}{\Vert {{}^{N} \underline{c}^{\,i}}_{n} + \varDelta ({{}^{N} \underline{c}^{\,i}}_{n}) \Vert } \end{array}\right. } \end{aligned}$$

The updated orientation of the collagen fiber inclusion can be directly calculated as

31 $$\begin{aligned} \begin{aligned}&{\phi ^{i}_{n+1}= {\left\{ \begin{array}{ll} \cos ^{-1}[{{}^\perp \underline{c}^{\,i}}_{n+1} \cdot \underline{K}_{\,n+1}] &{} \text {if } \sin [{{}^\perp \underline{c}^{\,i}}_{n+1} \cdot \underline{K}_{\,n+1}] \ge 0\\ 2\pi - \cos ^{-1}[{{}^\perp \underline{c}^{\,i}}_{n+1} \cdot \underline{K}_{\,n+1}] &{} \text {if } \sin [{{}^\perp \underline{c}^{\,i}}_{n+1} \cdot \underline{K}_{\,n+1}]<0 \end{array}\right. }}\\&{\theta ^{i}_{n+1}= \cos ^{-1}[{{}^{N} \underline{c}^{\,i}}_{n+1} \cdot \underline{T}_{\,n+1}]} \end{aligned} \end{aligned}$$

**Fig. 3:**
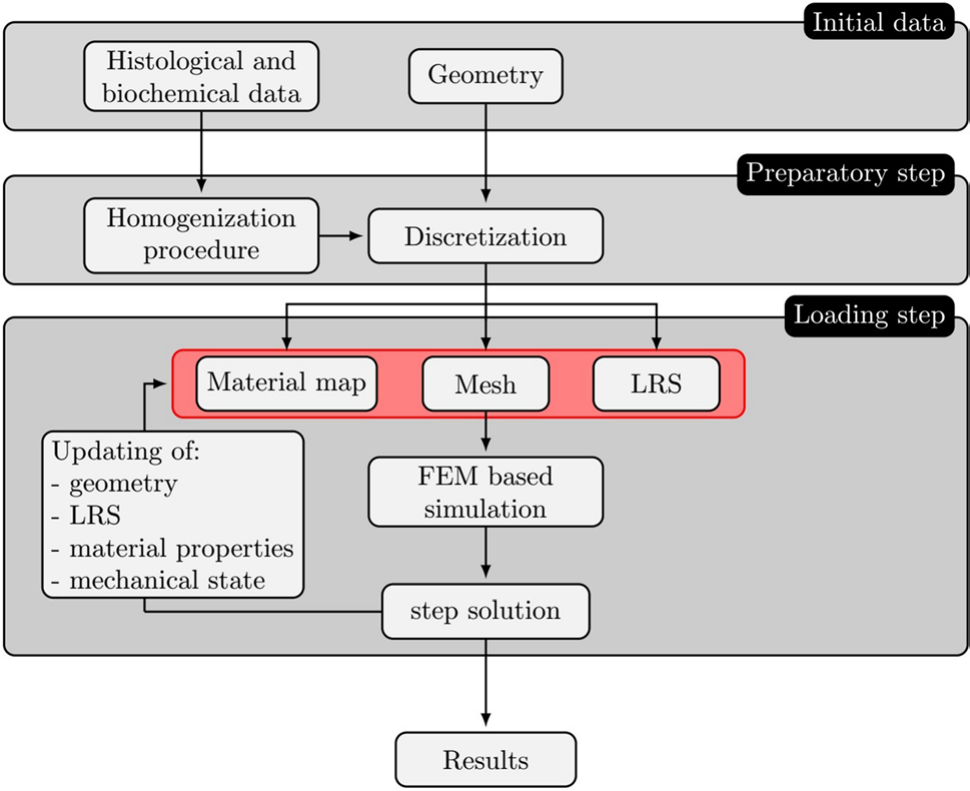
Flowchart of the solution algorithm based on a finite element implementation. LRS: local reference system; FEM: finite element method

In regard to the evolving stiffness of the collagen fibers, it is worth remarking that the modulus $E^{c,i}$ of the *i*th collagen fiber depends on the along-the-chord strain ${\varepsilon }^{i}$ whose increment is defined in the material reference system as

32 $$\begin{aligned} \varDelta {\varepsilon }^{i}_{n}={\underline{c}^{\,i}}_{n} \cdot \mathbf{d}^{c,\,i}_n \cdot {\underline{c}^{\,i}}_{n} \end{aligned}$$

resulting in a current strain in the fiber direction of ${\varepsilon }^{i}_{n+1} = {\varepsilon }^{i}_{n}+\varDelta {\varepsilon }^{i}_{n}$. With the updated chord elastic modulus, the macroscopic homogenized stiffness can be obtained. The overall algorithm is summarized in Fig. 3

## Case studies definition: validation on *P*-tests and *S*-tests

The presented model is validated by comparing its predictions to experiments performed on a cylindrical segment of carotid arteries from New Zealand White rabbits (Krasny et al. (2018)). To this aim, tension–inflation tests are simulated. According to the experiments, five loading scenarios are numerically addressed:

- two inflations under a constant axial stretch (*P*-test). The *P*-tests simulate the whole experimental process. Starting from the unloaded ex vivo configuration (on which the morphological parameters are determined), an axial stretch $\lambda$ is first applied on the structure, $\underline{U}^{{\rm im}}(t)=\underline{U}^{{\rm im}}=(\lambda -1)\,\mathcal {L}_o$; then, maintaining a constant stretch of either 1.5 or 1.8, a pressure ${P^{\,im}}(t)$ is monotonously applied from 0 to 140 mmHg.
- three axial tensions under constant imposed pressure (*S*-test). The *S*-tests simulate the whole experimental process. Starting from the fully unloaded ex vivo configuration (on which the morphological parameters are determined), an inner pressure ${P^{\,im}}$ is incrementally imposed on the structure, ${P^{\,im}}(t)=P^{\,im} \in \{ 20,100,140 \}$ mmHg ; then, maintaining the inner pressure constant at one of the three pre-cited values, an axial stretch $\underline{U}^{{\rm im}}(t)=\underline{U}^{{\rm im}}=(\lambda -1)\,\mathcal {L}_o$ is monotonously applied from a stretch of 1 to a 1.8 stretch.

**Fig. 4:**
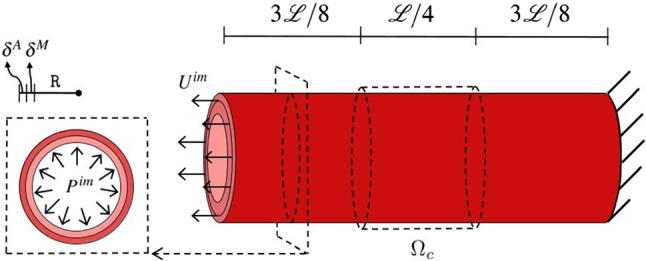
Representation of the case study: main geometrical features defining the computational domain and sketch of the boundary conditions

A sketch of the computational case study is provided in Fig. 4.

For all cases, the simulations incorporate four fiber families. Since they lie in the axial-circumferential plane, the angle $\phi$ is set to $\pm \frac{\pi }{2}$ (to keep the symmetry of the model) and the two angles $\theta$ are determined from image processing of the initial microstructure configuration (as measured by second harmonic generation). In more details, the angle probability density obtained from image processing is fitted by a sum of two Gaussian curves, and the highest peak is considered as the principal orientation of the fibers, labeled $\theta ^1$ in the sequel and reported first in Table 1. It is worth noting that the initial mechanical state of the tissues is reproduced in the simulation by defining the tissue model histological parameters directly measured from the microscopic analyses of the initial experimental configuration (Krasny et al. 2017, 2018) and with available data (Maceri et al. 2012, 2013; Marino and Vairo 2013; Morin et al. 2018; Khamdaeng et al. 2012). Values of parameters are summarized in Table 1.

**Table 1:** Values of model parameters employed in numerical simulations

Parameter	Symbol	Value in *S*-test	Value in *P*-test	References
Initial arterial segment length	$\mathcal {L}_o$ (mm)	7.8	8.0	Macro-measure from Krasny et al. (2018)
Initial arterial average radius	$\mathtt {R}_{o}$ (mm)	1.2	1.1	Macro-measure from Krasny et al. (2018)
Initial arterial thickness	$\delta _o$ (mm)	0.19	0.155	Macro-measure from Krasny et al. (2018)
Initial medial layer thickness	$\delta ^M_o$ (mm)	0.125	0.1	Image processing from Krasny et al. (2018)
Initial adventitial layer thickness	$\delta ^A_o$ (mm)	0.065	0.055	Image processing from Krasny et al. (2018)
Collagen volume fraction	$f^{\,c}$ (%)	15.0	20.0	Image processing from Krasny et al. (2018)
Initial collagen inclusion orientation vectors	${\theta _o}$ ($^\circ$) ${\phi _o}$ ($^\circ$)	$\{60,-60,10,-10\}$ $\{90,-90,90,-90\}$	$\{70,-70,35,-35\}$ $\{90,-90,90,-90\}$	Image processing from Krasny et al. (2018)
Initial fiber crimp amplitude	$h_o$	$0.2 \,\ell _o$	$0.25 \,\ell _o$	Image processing from Krasny et al. (2018)
Collagen fiber radius	$r^{\,c}$	$0.04\, \ell _o$	$0.04\, \ell _o$	Maceri et al. (2012, 2013); Bianchi et al. (2016)
Initial fiber period	$\ell _o$ ($\mu$m)	50.0	50.0	Image processing from Krasny et al. (2018)
Collagen Poisson's ratio	$\nu ^c$	0.35	0.35	Morin et al. (2018)
Matrix Young's modulus	$E^m$ (kPa)	10.0	10.0	Morin et al. (2018)
Matrix Poisson's ratio	$\nu ^m$	0.4	0.4	Morin et al. (2018)
Media Young's modulus	$E^M$ (MPa)	0.12	0.12	Khamdaeng et al. (2012)
Media Poisson's ratio	$\nu ^M$	0.49	0.49	Bianchi et al. (2016)
Collagen fiber Young's modulus	$E^f$ (MPa)	50.0	50.0	Marino and Vairo (2013); Bianchi et al. (2016)

The proposed integrated computational approach has been implemented via a parametric code developed in the Python environment (Python Software Foundation), exploiting finite element solver-core libraries of ABAQUS (ABAQUS / Standard; SIMULIA Inc, Providence RI). In particular, the code, solving the structural analysis at each discrete time instant $t_n$ by means of the ABAQUS libraries for mechanics (i.e., part, sketch, step, interaction, load, mesh, job, odbAccess), drives the enforcement of constitutive relations and the update of the reference systems for next time step $t_{n+1}$ via a post-processing of the results obtained at the previous step. The arterial domain is discretized by means of eight-node brick elements. As a result of a preliminary sensitivity analysis, an average mesh size equal to $\mathtt {R}_{o}/12$ is adopted. In regard to the discretization of the load and stretch steps, a sensitivity study was performed, and the step size of $\varDelta P =5$ mmHg and of $\varDelta \lambda =0.03$ (i.e., an incremental displacement of $|\varDelta \underline{U}^{{\rm im}} |=0.3$ mm) has been chosen for pressure load and stretch assuring a negligible discretization error less than 1%.

## Results and discussion

### Model validation at the fiber scale

**Fig. 5:**
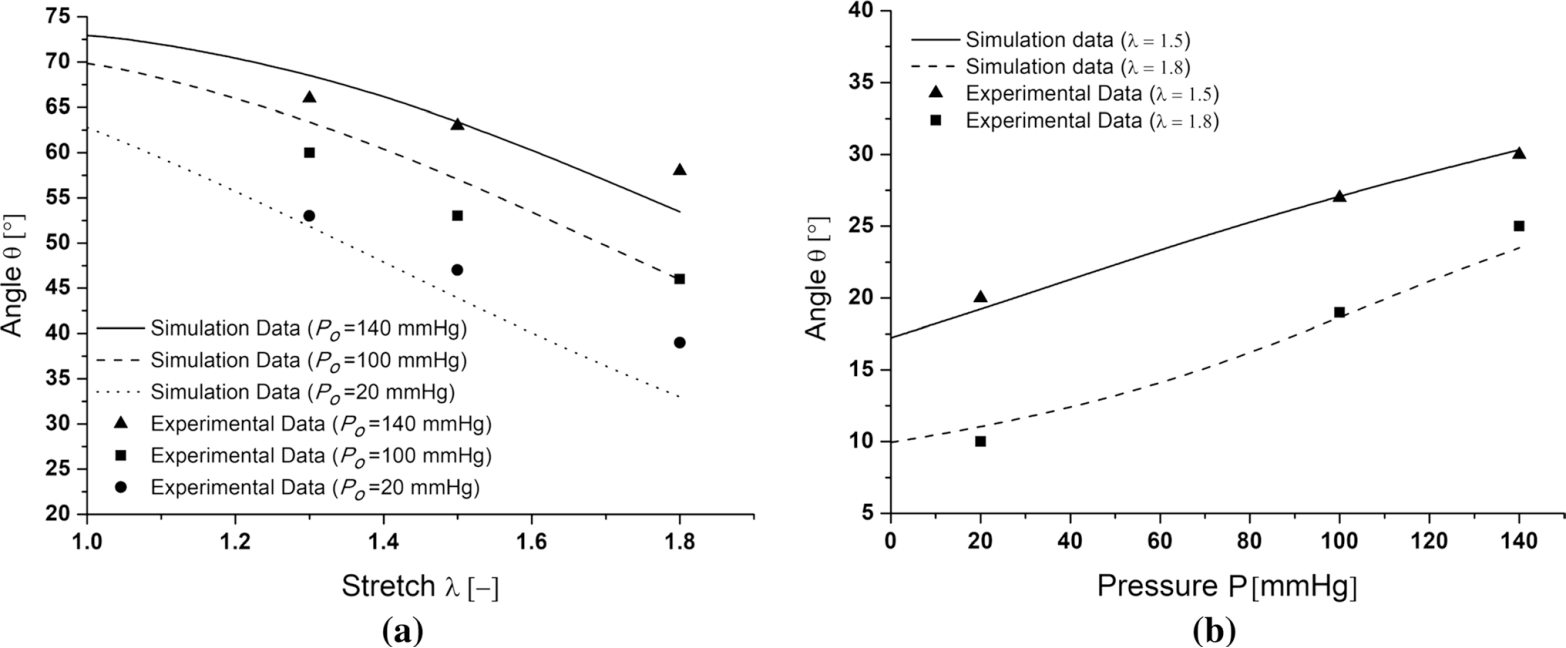
Orientation evolution of the main collagen family fiber during: the stretching simulation under different constant imposed pressure levels *S*-test (**a**); the inflation test under different constant axial stretch levels *P*-test (**b**)

The simulations of the tensile tests under constant pressure (*S*-test) and of the inflation tests at constant axial stretch (*P*-test) provide insight on the evolving fiber orientation with respect to the applied load, shown in Fig. 5 with comparisons to experimental data. More precisely, the average value of the first collagen fiber family, over the central region (with length $\mathcal {L}/4$ as shown in Fig. 4), is reported, in order to avoid boundary effects and to compare with experiments, in which the microstructure was imaged at the center of the samples. It is particularly interesting to note the very good agreement between the model prediction and the experimental data, given the fact that no angle adjustment was performed between different simulations. The maximum error on the angle prediction indeed amounts to 20% and is obtained at a stretch of 1.8 and a pressure of 20 mmHg.

**Fig. 6:**
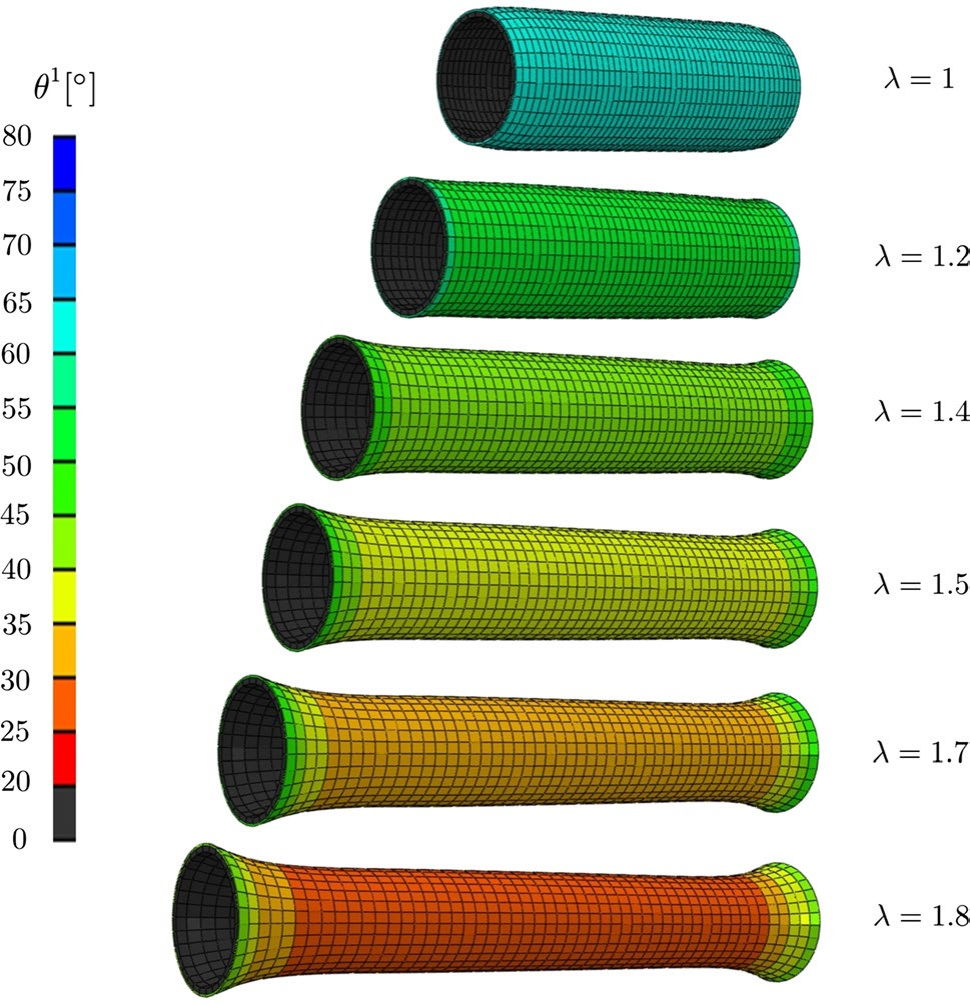
Spatial distributions of $\theta ^1$ angle, representing the orientation of the main family fiber 1 in the plane at different levels of stretch $\lambda$ imposing a constant pressure of $P^{\,im} = 20$ mmHg

Based on the definition of the $\theta$ angle, the 0 corresponds to the axial direction which, in turn, corresponds to the asymptote of the fiber orientation under axial stretch. However, the higher the inner pressure, the less reorientation is observed, since the circumferential stress prevents the rotation (Fig. 5a). On the other hand, the $\pi /2$ angle corresponds to the circumferential direction. It is interesting to see that the simulation well predicts the very limited reorientation occurring during inflation. This is a consequence of the large axial stretch imposed on the sample, which already produced a large reorientation toward the axial direction. Moreover, applying 140 mmHg does not produce a large stretching of the sample, which also explains the limited reorientation toward the circumferential direction. Furthermore, Fig. 6 provides the spatial distribution of the $\theta ^1$ angle of the main fiber family at different levels of stretch imposing a constant pressure $P^{\,im} = 20$ mmHg and it highlights the stretch-based re-orientation. As expected, because of the symmetry of the problem, the $\theta ^1$ keeps a constant value in the adventitial layer excepted at the ends of the segment due to the boundary conditions.

### Macroscopic mechanical response during tensile–inflation tests

**Fig. 7:**
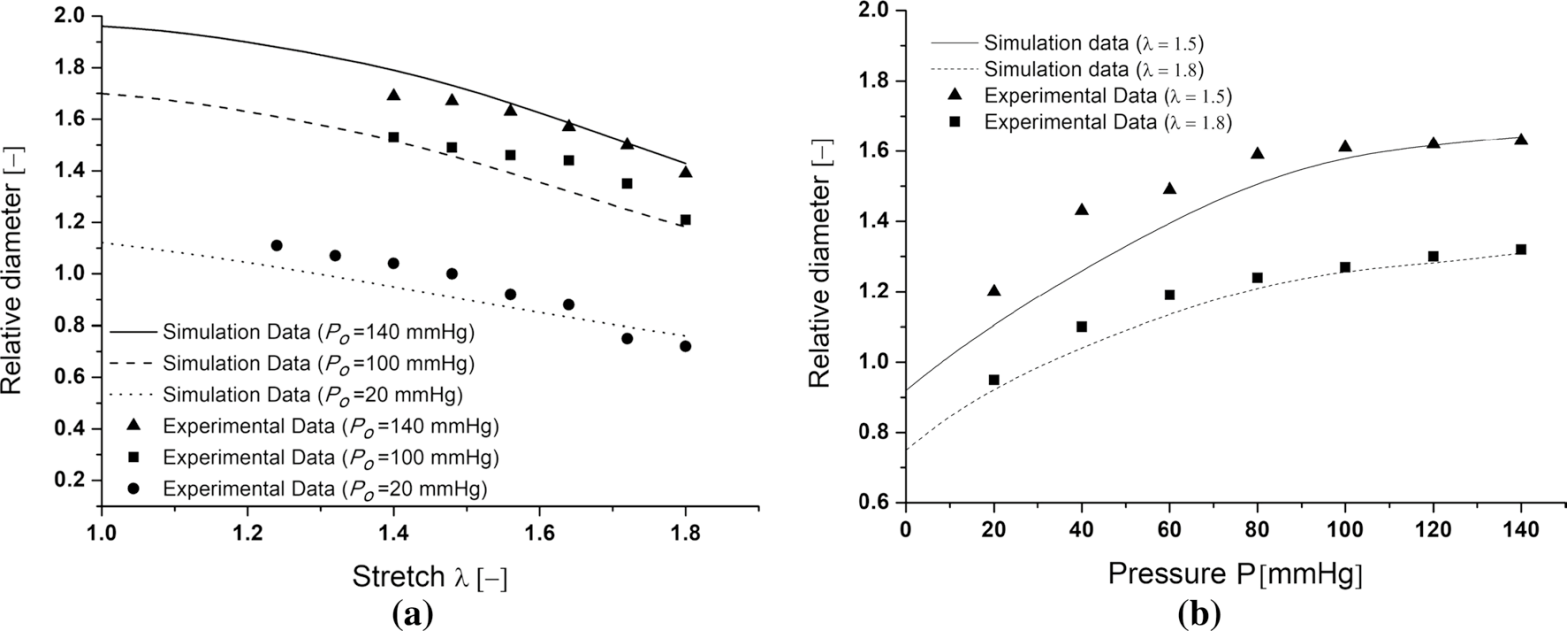
Mean radius evolution during: the stretching simulation under different constant imposed pressure levels *S*-test (**a**); the inflation test under different constant axial stretch levels *P*-test (**b**)

The simulations also provide access to the macroscopic mechanical response of the samples. Figure 7a reports the evolution of the circumferential stretch, computed as the average relative diameter over the central region, with respect to the applied axial stretch during the tensile tests. Similarly, Fig. 7b shows the evolution of the average relative diameter as a function of the applied pressure during inflation tests. For both series of tests, a very good prediction of the macroscopic kinematics of the samples is highlighted, as quantified by a maximum error of 15% between the model prediction and experimental data.

## Model capabilities analysis

### Collagen disorder investigation

**Fig. 8:**
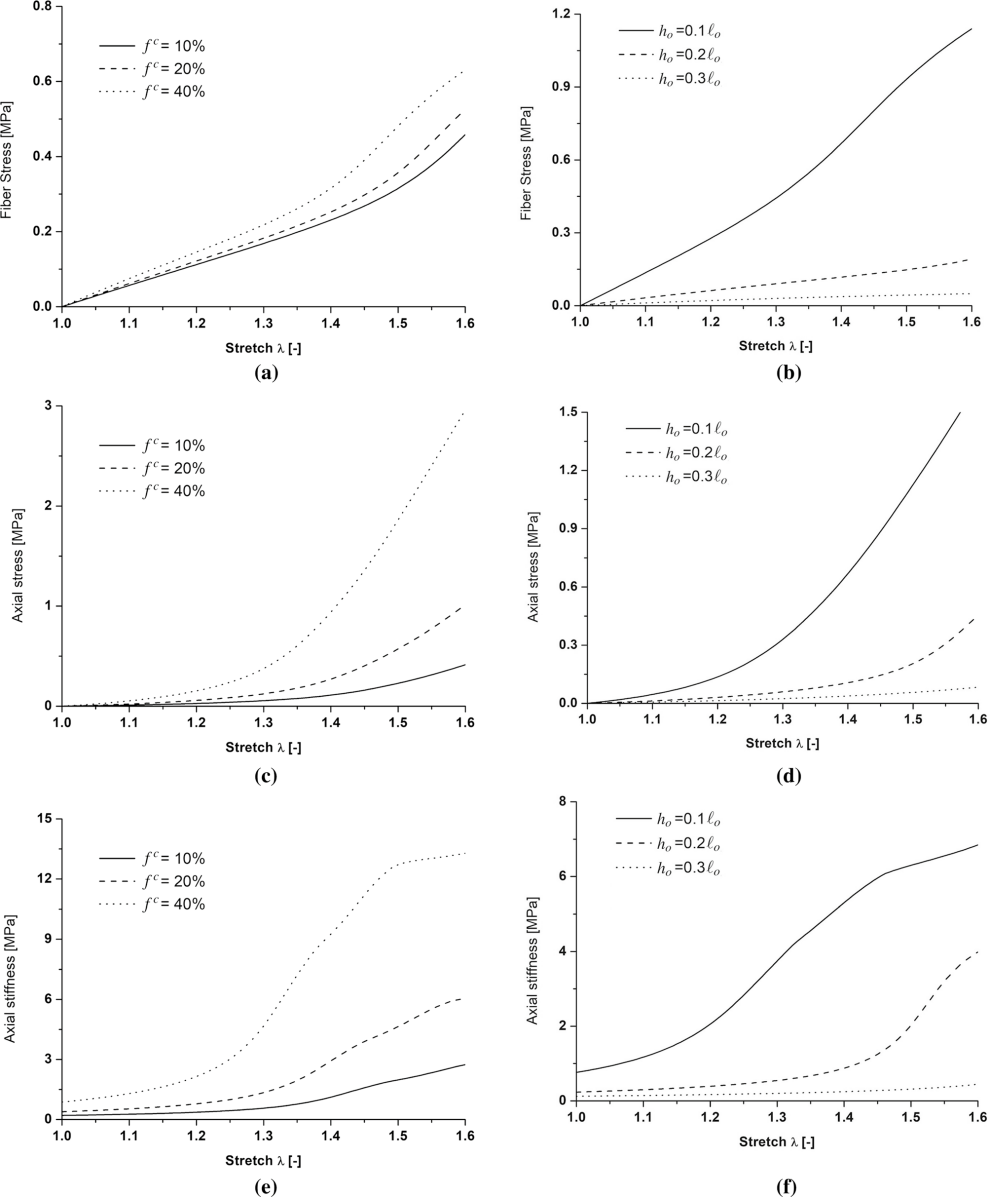
Influence of collagen volume fraction $f^{\,c}$ and of the collagen fiber crimp amplitude *h*, respectively, on: collagen fiber stress (**a**, **b**) , the axial stress of the arterial tissue (**c**, **d**) and the axial stiffness of the arterial tissue (**e**, **f**)

Several collagen-related alterations cause malfunction in the mechanical response of arterial tissues triggering remodeling mechanisms that can lead to arterial diseases (e.g., aneurysms). The adopted multiscale constitutive framework allows evaluation of the influence of the histological features on the macroscale tissue behavior as well as providing an insight into the micromechanical quantities that can represent the main features in the remodeling phenomena of the arterial tissues. For instance, some genetic disorders (such as the Ehlers–Danlos syndrome) are associated with a reduction in collagen content (Tsamis et al. 2013; Mao et al. 2002) leading to a higher risk of vascular rupture. Aiming at highlighting the behavior and the influence of collagen features on the mechanical response, using the parameters reported in Table 1 except for the histological features investigated, a stretching test is performed increasing the axial stretch (along the $\underline{T}$ direction) from $\lambda =1$ to $\lambda =1.6$ and imposing a constant pressure of $P^{\,im} = 20$ mmHg. Demonstrating the capabilities of the adopted constitutive model, Fig. 8 shows the influence of the collagen volume fraction $f^c$ and of the collagen fiber crimp amplitude *h* on the strain-induced evolution of macro- and micro-mechanical quantities adopting a range of values, respectively, of [10%, 20%, 40%] for $f^{\,c}$ and of $[0.1\,\ell _0$, $0.2\,\ell _0$ ,$0.3\,\ell _0]$ for *h*. The mean values of the mechanical quantities shown in Fig. 8 are averaged over the central region $\varOmega _c$ (Fig. 4) in order to avoid boundary effects. Figure 8a, c and e show the influence of the collagen volume fraction on the collagen fiber stress, the tissue stress, and stiffness, respectively. In this analysis, the variation in $f^{\,c}$ from 20 to 40% at stretch $\lambda =1.6$ causes a threefold increase in the axial stress and a twofold increase in the axial stiffness that corresponds to a 15% increase in fiber stress at the microscale. Moreover, Fig. 8b, d and f shows the influence of the collagen crimp on the same mechanical quantities highlighting different levels of the collagen fiber recruitment. In particular, for the value of $h_0=0.3\,\ell_0$, collagen fibers are still undulated, even at stretch $\lambda =1.6$, causing a lower stiffness in the arterial tissue.

**Fig. 9:**
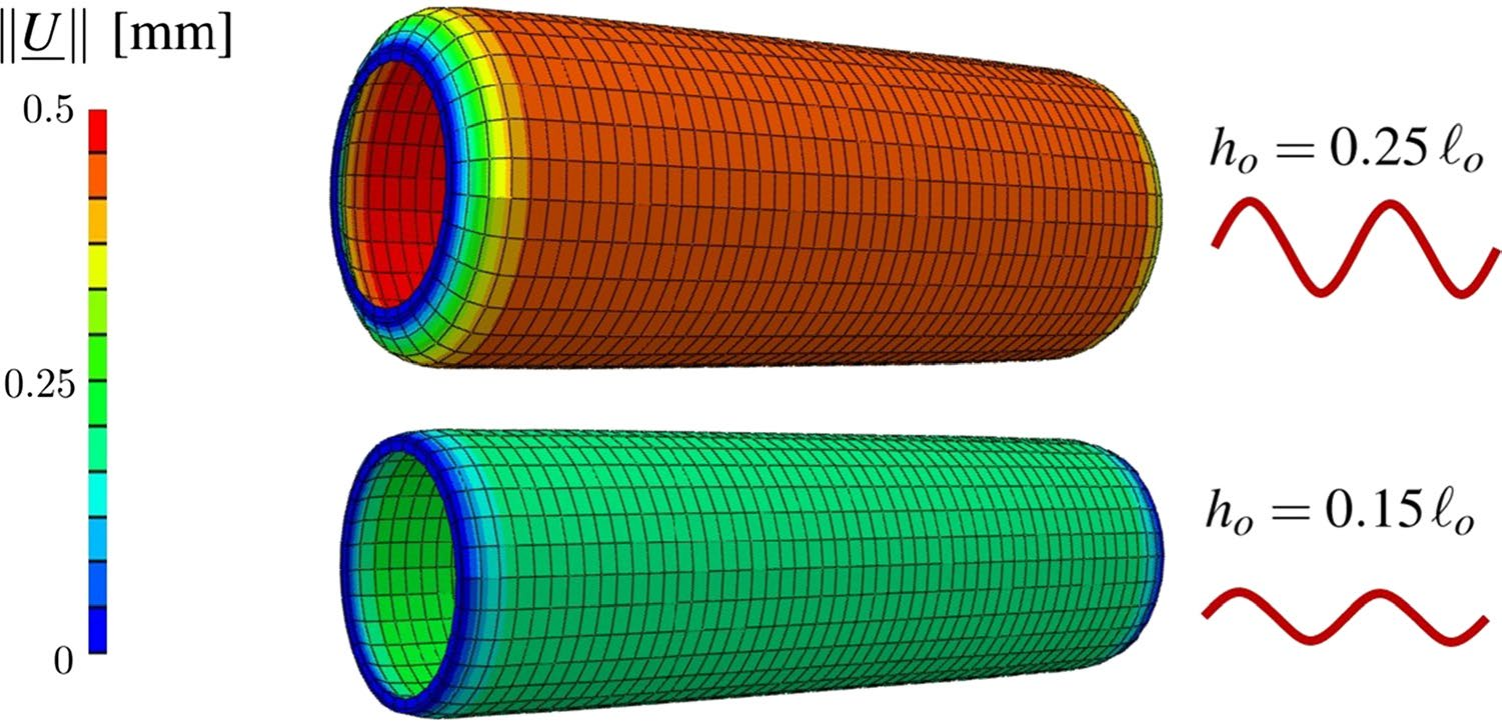
Spatial distribution of the displacement norm $\Vert \underline{U} \Vert$: influence of the initial fiber crimp amplitude $h_o$ on the displacement field imposing an internal pressure of $P^{\,im} = 140$ mmHg

Furthermore, Fig. 9 clearly illustrates the link between the histological features and the macroscopic mechanical response of the arterial tissue obtained thanks to the structural homogenization approach, with a *P*-test imposing a constant stretch of $\lambda =1.2$. The obtained results at a pressure $P^{\,im} = 140$ mmHg highlight the effect of the fiber crimp amplitude on the displacement field, with an increase in about two times in the displacement norm $\Vert \underline{U} \Vert$ when the crimp amplitude $h_o$ is increased by a factor of 0.7, demonstrating that the morphological features of the collagen fiber affect the mechanical properties of the tissue and, in turn, the mechanical response to the imposed pressure.

In conclusion, the presented results highlight that the proposed computational strategy is able to describe the strong coupling among macroscopic tissue constitutive response, non-affine deformation in the microstructure and local stress and/or strain field. In fact, Figs. 8 and 9 show the possibility to analyze the effects of interactions and crossed effects between different microstructural parameters, within the frame of known diseases.

### Relevance and contribution

In the framework of arterial constitutive modeling, although several effective methods exist to model the mechanical behavior of the arterial tissues (Holzapfel et al. 2000; Hollander et al. 2011), they generally exhibit a weak relationship between model parameters and tissue histological/biochemical features (though often inspired from those). This prevents from any possibility to understand if a specific biological or histological feature of the tissue can contribute in a mechanical response. In fact, a phenomenological model foregoes any attempt to explain why the variables interact, and simply attempts to describe the response. Instead, the main objective of the developed model is to give an insight into micromechanics of the arterial tissues and, as a prospect, into the mechanism that can drive tissues to pathological remodeling. For this reason, the parameters in our mechanistic model have biological definitions and the hypothesized relationship between the variables derives from the description of the nature of the relationship in terms of the mechanical and biological processes. This fundamental aspect allows our model to investigate how a specific histological feature contributes to the micro- or macro-mechanical environment of the tissue. Moreover, this capability naturally extends our model for the investigation of microstructural remodeling. In addition, with respect to other structurally motivated constitutive models presented in the literature (Marino and Vairo 2013; Maceri et al. 2010, 2013), the developed model reproduces the non-affine kinematics of the collagen fibers allowing the model to analyze the real mechanical environment of the fiber and of the arterial tissue. In fact, the microscopic kinematics represents a key aspect of the constitutive modeling in soft tissues and many recent experimental evidences in mechanics of the biological tissues, especially when load bearing is primarily handled by a fiber network, highlight that the assumption of affine fibers movement with macroscopic deformation is not accurate. In particular, the experiments show that each fiber is free to move inside the soft matrix and the fiber strain is contained by an extensive re-orientation (Cavinato et al. 2020; Krasny et al. 2017; Chandran and Barocas 2006). In the developed model, the non-affine kinematics of the fibers is naturally taken into account.

**Fig. 10:**
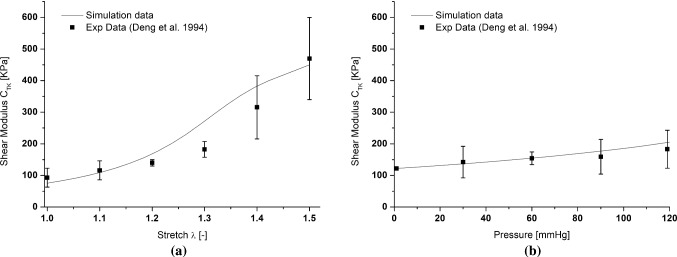
Evolution of the shear stiffness between axial and circumferential directions during: the stretching simulation under constant imposed pressure $P = 120$ mmHg (**a**); the inflation test under constant axial stretch levels $\lambda = 1.3$ (**b**). Comparison with experimental data (Deng et al. 1994)

Additionally, the proposed constitutive model is able to predict experimental evidences which are hardly dealt with in the literature. For example, the work of Deng et al. (1994) shows the evolution of the shear modulus in relation to stretch and pressure of the arterial tissue. The experimental results have been used highlighting the ability of the model to reproduce the coupling observed concerning the shear stiffness. In particular, Fig. 10 shows the comparison between the evolution of the shear stiffness between axial and circumferential directions measured in (Deng et al. 1994) and the simulated one.

Figure 10a, b shows the evolution of the shear stiffness during a stretching test maintaining a constant imposed pressure of 120 mmHg and a pressurization test maintaining a constant stretching level $\lambda = 1.3$, respectively. This comparison highlights the good agreement regarding the coupling between shear stiffness and stretch of the tissue. In fact, an increase in longitudinal (i.e., stretching test) and circumferential (i.e., pressurization test) stretch level corresponds to an increase in the shear stiffness. To get these results, the contribution of Deng et al. (Deng et al. 1994) did not report the histological data of the samples necessary to retrieve the material parameters. Therefore, for this specific case, the comparison is made using a model parameter in agreement with the literature for arterial tissues (Morin et al. 2018; Bianchi et al. 2016; Marino and Vairo 2013; Khamdaeng et al. 2012).

## Conclusion

This paper presents a novel constitutive model that captures the complexity of load-induced micro-structural morphological changes and links the mechanical response of the tissue with the histological features of the constituents. Moreover, the proposed computational approach implemented in a finite element-based numerical tool can be employed in numerical simulations reproducing any mechanical test and can be straight forwardly applied to a patient-specific framework.

The computational model has been validated on the basis of experimental tests reproducing microscopic and macroscopic mechanical behavior of the arterial tissue. In detail, using values of model parameters fully consistent with histological and morphological experimental evidence, numerical simulations addressing different mechanical tests have been compared with experimental results proving effectiveness and accuracy of the proposed multiscale approach.

Moreover, the utility of the model to locally evaluate histological features and the microstructure of the arterial tissue during the physiological loading can elucidate local mechanical stimuli affecting tissue cellular environment in pathological remodeling, enabling key insight on pathogenesis and progression.

Future works will address the microstructure of the medial layer considering the collagen and elastin fiber inclusions and will take into account damage and viscous effects occurring in the arterial tissue at different scales. Furthermore, in order to obtain useful results for clinical application, patient-specific geometries will be investigated including density distributions for the orientation of the fibers (e.g., collagen, elastin). Moreover, exploiting the flexibility of the computational approach based on FE, the transmural effects (e.g., the stress variation inside the tissues) will be analyzed.
